# Does artificial intelligence-supported rhythm-enhanced phonological awareness training improve early reading in children with attention-deficit/hyperactivity disorder? A randomised controlled intervention study

**DOI:** 10.3389/fpsyg.2026.1906514

**Published:** 2026-09-03

**Authors:** Linzi Zhang, Fangya Huang, Panpan Li

**Affiliations:** 1 Department of English, Hainan University, Haikou, China; 2 School of International Communication and Arts, Hainan University, Haikou, China; 3 School of International Studies, Hainan University, Haikou, China

**Keywords:** ADHD, AI, phonological awareness, reading, rhythm

## Abstract

**Introduction:**

Children with attention-deficit/hyperactivity disorder (ADHD) are at increased risk of reading-related difficulties, and phonological awareness (PA) plays a vital role in early reading development. Rhythm may serve as a temporal scaffold for PA, yet whether rhythm-enhanced PA training produces additional benefits beyond typical PA training for children with ADHD remains unclear. This study examined whether rhythm-enhanced PA training, delivered through an artificial intelligence (AI)-supported and research-assistant-facilitated interactive teaching model, could improve early reading-related abilities and rhythm discrimination in Mandarin-speaking kindergarten children with ADHD.

**Methods:**

A total of 165 eligible kindergarten children with ADHD were stratified by gender and randomly allocated in a 1:1:1 ratio to the rhythm-based phonological awareness (RPA) intervention group, the phonological-awareness-only (PAO) intervention group, or the control group. Of these, 146 children completed the intervention and were included in the final analysis. Both intervention groups received AI-supported instruction for 8 weeks, with two 30-min sessions per week, while the control group received AI-supported oral storybook activities. Outcome measures included character reading, four PA measures (syllable, rhyme, tone, and phoneme-level awareness), and rhythm discrimination.

**Results:**

Significant time × group interactions were found for character reading, all four PA measures, and rhythm discrimination (all *p*s < 0.001, $\eta_{p}^{2}$ = 0.11–0.53). Bonferroni-adjusted comparisons showed that both intervention groups outperformed the control group on syllable, rhyme, tone, and phoneme-level awareness (*p*s < 0.05). The RPA group further outperformed the PAO group in character reading (mean difference = 6.92, *p* = 0.003), syllable awareness (mean difference = 1.72, *p* = 0.004), rhyme awareness (mean difference = 1.54, *p* < 0.001), and rhythm discrimination (mean difference = 3.11, *p* < 0.001).

**Discussion:**

Within the shared AI-supported and human-facilitated interactive teaching model, PA intervention improved PA-related outcomes, while rhythm enhancement provided additional benefits for character reading, syllable awareness, rhyme awareness, and rhythm discrimination. This study further extends the Temporal Sampling Framework by demonstrating that rhythm may serve as a temporal scaffold for PA and early reading development in children with ADHD.

## Introduction

Phonological awareness (PA) plays a vital role in early reading development, enabling children to segment, identify and manipulate speech sounds that are later mapped onto letters and used in word decoding (Ahokas et al., 2025a, 2025b; Dong et al., 2020). However, children with attention-deficit/hyperactivity disorder (ADHD) are at increased risk of reading-related difficulties, although the nature and severity of these difficulties vary across children and are often more pronounced when reading disability co-occurs (de Groot et al., 2017; Dong et al., 2023; Caldani et al., 2022; Miao et al., 2026). In addition, PA activities require children to sustain attention, retain speech-sound units in working memory, inhibit irrelevant responses and repeatedly manipulate similar phonological forms (Cole et al., 2023; Shen et al., 2025; Ruan et al., 2024; Zhang et al., 2026). These demands may reduce task persistence and engagement among children with ADHD. Moreover, Traditional typical PA interventions usually depend on repeated drills, prompt-based practice and continuous corrective feedback (Shen et al., 2025; Ruan et al., 2024; Zhang et al., 2026). Although such techniques are theoretically appropriate, they may be less efficient for children with ADHD, who are more likely to experience reduced task persistence, attentional fluctuation and disengagement during repetitive learning activities (Dong et al., 2026; Gutiérrez-Palma et al., 2026; Miao et al., 2026).

Past studies have highlighted rhythm as a critical bridge between auditory processing and language development. The Temporal Sampling Framework (TSF; Goswami, 2011) posits that rhythmic processing enhances PA development. However, most rhythm-based reading or PA interventions have focused on typically developing children. In comparison, the contribution of rhythm-based PA (RPA) reading on the language development of children with ADHD remains unclear. Furthermore, whether rhythm-enhanced PA training produces additional benefits beyond typical PA training remains unclear.

AI-based systems may support the standardised delivery of intervention content by providing consistent pacing, prompts, target items, and feedback across sessions (Dong et al., 2023, 2026; Miao et al., 2026). However, technology-supported interventions involving young children commonly require human facilitation for device operation, attention management, participation support, and behavioural management (e.g., Yuan et al., 2026). In addition, only a few studies have investigated whether AI could supported RPA training for children with ADHD. In particular, it remains unclear whether RPA training provides benefits beyond typical PA training for children with ADHD. Therefore, the current study extends the literature by examining the effects of AI- supported RPA intervention on the language development of children with ADHD. In the present study, the AI chatbot delivered all instructional content, including oral instructions, target words, instructions for task actions, rhythmic cues, task pacing and corrective feedback. The trained research assistants (RAs) which played the role of facilitators, provided technical and non-instructional behavioural support only, including device operation, standardised attention redirection and implementation-fidelity recording. RAs did not explain or rephrase instructional content, demonstrate task actions, provide hints or corrective feedback, evaluate children’s responses or otherwise scaffold task completion. In this approach, AI is used as a standardised delivery tool that may reduce instructor-related variability and ensure consistent implementation across sessions.

## Literature review

### Rhythm and children’s language development

The ability to follow speech rhythm has been identified as a key factor enabling children to learn how to read (Goswami, 2011). Once the rhythm of one’s own language is known, it is easier to pay attention to the acoustic signal and identify words and key elements that distinguish certain words from others, such as phonemes, syllables or rhymes (Goswami, 2011). The current literature suggests that spoken language is not processed as a sequence of isolated phonemes only. Rather, children also rely on rhythmic regularities in the speech stream to segment words, identify syllabic structures and build stable phonological representations (Ruan et al., 2024; Shen et al., 2025; Zhang et al., 2026). The TSF posits that children’s sensitivity to low-frequency amplitude modulations in speech may support the perception of syllables, stress patterns and larger prosodic units, thereby providing an auditory foundation for PA and early reading development (Goswami, 2011). Thus, rhythm may serve as an upstream auditory element and a phonological mechanism that helps children attend to the temporal structure of speech before they manipulate smaller phonological units, such as onsets, rhymes and phonemes.

However, existing findings in the literature are not fully consistent. On the one hand, some studies have shown that rhythm sensitivity is associated with children’s PA and related early language literacy development (Harrison et al., 2018; Holliman et al., 2008; Power et al., 2013). For example, Holliman et al. (2008) suggested that sensitivity to speech rhythm had a significantly positive association with children’s PA. Harrison et al. (2018) found that 4- to 5-year-old children receiving speech-rhythm training showed gains in word reading compared with a control condition. In contrast, Gutiérrez-Palma et al.’s (2026) study showed that rhythm training did not enhance PA development among children with reading difficulties.

### Effects of AI chatbot-supported intervention on children’s language development

Artificial intelligence chatbots have increasingly been incorporated into technology-supported language-learning activities for children (Cheng et al., 2024; Dong et al., 2026; Yuan et al., 2026). AI chatbots can respond to children’s answers, provide prompts, adjust feedback and maintain interactive exchanges during learning activities (Dong et al., 2026; Miao et al., 2026; Yuan et al., 2026). Sociocultural theory suggests (John-Steiner and Mahn, 1996) that children’s language development is shaped through guided participation and scaffolded interaction with more knowledgeable partners. In this sense, AI chatbots may function as effective instructional partners that support children’s engagement in PA activities, such as identifying, segmenting, blending and manipulating speech sounds.

Beyond language learning, AI-enabled systems have increasingly been used as components of intervention delivery in broader pediatric and educational contexts. AI-driven intelligent tutoring systems have been applied in K–12 education to provide structured instruction, practice and feedback, with generally positive but comparison-dependent effects on learning outcomes (Dong et al., 2026; Létourneau et al., 2025; Yuan et al., 2026). AI-based interventions have also been used to support students with learning disabilities, although the available studies vary considerably in their methodological quality and intervention design (e.g., Paglialunga and Melogno, 2025). In pediatric and family contexts, conversational agents have been used to deliver parenting interventions and other forms of structured guidance, with existing evidence supporting their feasibility and acceptability while indicating a need for stronger effectiveness studies (e.g., Klapow et al., 2024). AI-assisted intervention has also been examined directly among children with ADHD, including the use of AI-generated feedback in language and related emotional outcomes (Dong et al., 2026; Miao et al., 2026).

Past studies also suggested that PA learning requires structured instructional conditions in which children receive repeated opportunities to detect, compare and manipulate PA units. For example, Ehri et al. (2001) reported that PA instruction significantly enhanced children’s reading ability development, particularly when instruction is explicit and systematically delivered. Furthermore, PA intervention has been shown to improve children’s PA and word reading development (Ruan et al., 2024; Shen et al., 2025; Zhang et al., 2026).

During language intervention, AI chatbots may support the consistent delivery of target words, task prompts, rhythmic cues, pacing and corrective feedback across sessions (Liu et al., 2022; Miao et al., 2026; Yuan et al., 2026). Such consistency may be particularly relevant to PA intervention because variations in oral prompts, feedback timing and task pacing may affect children’s perception of phonological contrasts. However, existing pediatric and educational applications of AI have primarily focused on general learning support, reading engagement, family guidance or behavioural and therapeutic outcomes. Less is known about whether an AI-supported delivery model can be used to implement a theoretically specified and skill-focused PA intervention for clinically diagnosed children with ADHD. It also remains unclear whether rhythm provides additional benefits when PA content, dosage, task order and human facilitation are held constant.

### Phonological awareness learning in children with ADHD

ADHD is a neurodevelopmental disorder typically diagnosed during childhood (Dong et al., 2026; Miao et al., 2026), its estimated worldwide prevalence is approximately 5.3% among children and adolescents and 2.5% among adults (Polanczyk et al., 2007; Simon et al., 2009). The preschool years represent a critical time for children with ADHD to develop their language skills that they need to succeed in school (Dong et al., 2023; Leonard et al., 2009; Niu et al., 2021). Children with ADHD usually experience learning difficulties and psychological distress due to attentional disorders (Dong et al., 2023; Niu et al., 2021). They may also face particular challenges in PA learning, because PA tasks often require listening carefully to spoken stimuli, holding sound units in working memory, inhibiting irrelevant responses, comparing similar phonological forms and repeating sound-manipulation tasks over multiple trials. While the current literature suggests that explicit PA instruction enhances early reading development, research on ADHD-specific PA intervention remains limited.

Children with ADHD are at increased risk of difficulties in early reading development, and PA represent important instructional targets for children who experience such difficulties. The current literature suggested PA tasks require children to listen carefully to spoken stimuli, retain sound units in working memory, inhibit irrelevant responses and repeatedly manipulate similar phonological forms. These task demands may create additional challenges for children with ADHD because of their difficulties in sustaining attention and regulating task engagement. Past intervention studies further indicated that improving ADHD symptoms alone may not be sufficient to address reading difficulties. Direct reading interventions targeting word reading, decoding and phonemic processing have been found to improve reading-related outcomes among children with ADHD and word-reading difficulties (Denton et al., 2020; Tamm et al., 2017). For example, Chan et al. (2023) conducted a meta-analysis to show that structured reading interventions can improve reading outcomes among at-risk readers with ADHD, particularly when instruction directly targets PA. These findings support the use of explicit phonological awareness instruction for children with ADHD and provide a basis for examining how such instruction can be delivered in a structured and engaging manner.

One promising intervention direction is to combine PA instruction with rhythm-based support. Rhythm may help children attend to the temporal structure of speech, thus making syllables, rhymes and phonological patterns more salient. For children with ADHD, rhythmic cues may also provide external temporal structure that supports attention, response timing and task engagement during repeated PA practice. However, to date, whether rhythm-enhanced PA training produces stronger effects than typical PA training for children with ADHD has yet to be fully investigated.

### The current study

This study examined whether structured PA intervention delivered through a shared AI-supported and RA-facilitated interactive teaching model improved PA-related outcomes in kindergarten children with ADHD, and whether adding rhythm produced benefits beyond PA-only instruction. The same delivery model was used across the RPA, PAO and oral-storybook control conditions. Therefore, the primary comparisons concerned differences in intervention content rather than the independent effect of AI relative to human-led delivery. The study addressed the following two research questions (RQs):

*RQ1*: Do RPA and PAO interventions delivered through a shared AI-supported, human-facilitated interactive teaching model produce greater gains in PA-related outcomes than an oral-storybook control condition delivered through the same model? It was hypothesised that both PA intervention groups would show greater gains than the control group on PA measures.

*RQ2:* Does adding rhythm to PA intervention produce additional benefits beyond PA-only intervention within the same delivery model? It was hypothesised that the RPA group would show greater gains than the PAO group on selected reading-related, PA, and rhythm outcomes.

## Methods

### Participants

Participants were recruited using convenience sampling from 27 kindergartens in one city in China that were accessible to the research team. All 27 kindergartens approached by the research team agreed to participate. Online recruitment posters were distributed through the participating kindergartens’ parent communication platforms to the parents or guardians of all 4,651 enrolled children. A total of 227 parents or guardians responded and reported that their children showed possible signs of ADHD. These children were subsequently invited to undergo formal diagnostic and eligibility assessment conducted by a licensed psychologist. Children were excluded if they had an identified comorbid psychiatric, neurodevelopmental, neurological disorder that could independently affect their participation in or response to the intervention. The excluded conditions included eating disorders, autism spectrum disorder, anxiety disorders, and neurological disorders. Comorbidity status was determined on the basis of a licensed psychologist’s clinical assessment and parent-reported medical and developmental history.

ADHD diagnosis and comorbidity screening were conducted by a licensed psychologist in accordance with the DSM-5 criteria. The assessments also included the Conners’ Parent Rating Scales to corroborate ADHD symptoms, the Autism Behaviour Checklist (ABC) to screen for autism spectrum disorder-related symptoms, and the Screen for Child Anxiety Related Emotional Disorders (SCARED) to screen for anxiety symptoms. The results of these standardised instruments were interpreted together with the psychologist’s clinical assessment and parent-reported medical and developmental histories, including available records provided by the parents. Children were excluded if an identified co-occurring psychiatric, neurodevelopmental, or neurological condition could independently affect their participation in or response to the intervention. The identified exclusionary conditions included eating disorders, autism spectrum disorder, anxiety disorders, and neurological disorders.

Of the 227 children who entered the diagnostic and eligibility assessment, 11 did not meet the DSM-5 diagnostic criteria for ADHD and 51 were excluded because they had identified exclusionary comorbid conditions. The remaining 165 eligible children met the DSM-5 diagnostic criteria for ADHD and had no identified exclusionary comorbid conditions. To achieve comparable group sizes and gender distributions, the children were first stratified by gender. Following random allocation, all children completed the pretest before the formal intervention. Within the boy and girl strata, the children’s names were entered into an online random number generator to generate a random sequence. They were then allocated in a 1:1:1 ratio to the rhythm-based phonological awareness (RPA) intervention group, the phonological-awareness-only (PAO) intervention group, or the control group, with 55 children initially assigned to each group. During the intervention period, 19 children withdrew because of unforeseen circumstances, including temporary medical circumstances unrelated to the pre-enrolment comorbidity criteria or moving to another city. These withdrawals occurred after the pretest and randomisation and were unrelated to the comorbidity-based exclusion criteria. The final analytic sample therefore consisted of 146 children, and their data were included in the analysis (RPA: 39 boys, 10 girls, Mean age = 5.55 years old, SD = 0.30; PAO: 34 boys, 14 girls, Mean age = 5.46 years old, SD = 0.26; control group: 39 boys, 10 girls, Mean age = 5.53 years old, SD = 0.31).

### Measures

#### Nonverbal intelligence

The present study used the short version of Raven’s (1995) Progressive Matrices Test (Sections A–C) to measure the selected children’s nonverbal intelligence. There were 36 items grouped into three sections, and the children were required to select one piece from the given options to best complete the given picture. Each correct answer was given a score of 1, and the maximum score was 36. The Cronbach’s *α* for this study was 0.88.

#### Working memory

The 18 item digit-span test was used to measure the children’s verbal working memory. In particular, the children were required to repeat digits orally forward and backwards. Three to seven digits were presented with increasing difficulty level. Each level contained three items, and the children were required to repeat the reverse order of the presented digits. Each correct answer was given a score of 1, and the maximum score was 18. The Cronbach’s α for this study was 0.85.

#### Receptive vocabulary

This study used the Chinese Receptive Vocabulary Test (Xu et al., 2024) to measure the selected children’s Chinese receptive vocabulary. The children were required to select one picture that best matched the semantic meaning of the presented words by the instructor. Every correct answer was given 1 point, and the maximum score was 60. The Cronbach’s α for this study was 0.93.

#### Character reading

Kim et al.’s (2020) Chinese Character Reading Test was used in the present study to measure the participants’ word reading ability. The children were asked to voice out each word presented by the instructor, and each correct answer was given 1 point. The test stopped when a child failed to read 15 words consecutively. The maximum score was 120, and the Cronbach’s α was 0.90.

#### Phonological awareness

To measure the children’s PA performance, the present study used a Mandarin PA task (Shu et al., 2008), which included four subtests: syllable awareness, rhyme awareness, tone awareness and phoneme-level awareness. In the syllable awareness task, the children were required to segment or delete a target syllable from Mandarin words presented to them orally. The syllable awareness subtest consisted of 16 items. For example, children heard the word ‘苹果 /ping 2 guo 3/(apple)’ and were asked to say what remained after deleting the syllable /ping 2/; the correct answer was /guo 3/. In the rhyme awareness task, the children listened to three orally presented words and selected the one that did not rhyme with the others. The rhyme awareness subtest consisted of 10 items. For example, the children heard three Mandarin words, such as mao /mao 1/, bao /bao 1/ and shu /shu 1/ and were asked to identify the word that did not rhyme with the others; the correct answer was shu /shu 1/.

In the tone awareness task, the children listened to three Mandarin syllables with the same segmental structure but different lexical tones and were asked to identify the item with a different tone. The tone awareness subtest consisted of 10 items. For example, the children heard three syllables with the same segmental structure but different lexical tones, such as /ma 1/, /ma 1/ and /ma 3/ and were asked to identify the syllable with a different tone; the correct answer was the third syllable /ma 3/. In the phoneme-level awareness task, the children were asked to identify or delete the initial sound from orally presented words. Finally, the phoneme-level awareness subtest consisted of 16 items. For example, the children heard the syllable /ma 1/ and were asked to say what remained after deleting the initial phoneme /m/; the correct answer was /a 1/. Each correct answer was given 1 point, and incorrect or no responses were given 0 points. The maximum scores for syllable awareness, rhyme awareness, tone awareness and phoneme-level awareness were 16, 10, 10 and 16, respectively. The Cronbach’s *α* values for syllable awareness, rhyme awareness, tone awareness and phoneme-level awareness were 0.87, 0.90, 0.82 and 0.86, respectively, for this study.

#### Rhythm discrimination

The task was a revised version of Wang and Chen’s (2025) musical rhythm discrimination task to examine children’s musical rhythm perception. The musical rhythm discrimination task contained 20 items driven by E-prime 1.0. The stimuli were presented auditorily using metronome-like tones to minimise the involvement of speech-specific phonological processing. The two rhythmic sequences in each trial were separated by an interval of approximately one second. In each trial, the children listened to two short rhythmic sequences and were asked to judge whether the two sequences were the same or different. The rhythmic sequences were presented auditorily, and the task did not require children to produce motor responses, such as clapping or tapping. Two practice trials were conducted before the formal test to ensure that children understood the task requirements. Each correct response was scored as 1, and each incorrect or missing response was scored as 0. A higher score indicated better rhythm discrimination ability. The maximum score for the musical rhythm discrimination task was 20, and the Cronbach’s α was 0.91.

### Research procedure

All research procedures involving human participants in this randomised controlled intervention study were reviewed and formally approved by the Ethics Committee of Guangzhou Huashang College (ethics approval no. HSEDU20240092; approval date: December 12, 2024). Prior to conducting the tests, we collected the assent and consent forms from the children and their parents, respectively. The research team provided a demographical questionnaire to parents and then administered pretests to the children to evaluate their nonverbal intelligence, working memory, receptive vocabulary, character reading, PA and rhythm discrimination. Before the intervention, we recruited 30 research assistants (RAs), who were selected on the basis of their successful related research experiences.

The 30 RAs received a two-hour training workshop and completed one supervised practice session. The training covered the intervention rationale, task procedures, standardised prompts, child-attention management, fidelity checklists and ethical procedures. The RAs were required to follow the intervention manual, which explicitly limited their role to technical and non-instructional behavioural support. The RAs were permitted to operate or troubleshoot the device, ensure that the children could hear and view the chatbot, use standardised non-instructional reminders to redirect the children’s attention to the chatbot and complete the implementation-fidelity checklist. The RAs were not permitted to explain or rephrase instructional content, demonstrate task actions, provide hints or corrective feedback, evaluate children’s responses or otherwise scaffold task completion. These role boundaries were applied consistently across the RPA, PAO and control groups. A hard copy of the detailed guidelines for implementing the RPA, PAO or control condition using the AI chatbot was provided to each corresponding RA.

The children from the three groups completed the intervention in separate rooms and at different time slots. The intervention lasted for 8 weeks, with two 30-min sessions per week, resulting in 16 sessions and a total planned dosage of 480 min. To our knowledge, no published clinical or intervention-design guideline specifically prescribes an optimal dosage for AI-supported PA intervention in kindergarten children with ADHD. The intervention schedule was therefore informed by evidence from related child language interventions. A previous reading intervention with kindergarten children with ADHD used approximately 25-min sessions twice per week, providing an age- and population-relevant precedent for the twice-weekly frequency and approximate session length adopted in the present study (Dong et al., 2023). In addition, a 12-week AI chatbot-supported shared-reading intervention demonstrated the feasibility of sustained AI-mediated language intervention in kindergarten children with ADHD, although it did not establish the optimal dosage for the present intervention (Dong et al., 2026). Moreover, the total planned dosage of 8 h falls within the 5–18-h range associated with larger effects of phonemic awareness instruction in a previous meta-analysis (Ehri et al., 2001). Thomson et al. (2013) suggested the 6-week rhythmic and PA intervention would be an appropriate intervention duration for language development amongst children with developmental dyslexia. Miao et al. (2026) implemented a 4-week AI-assisted parent–child dialogic-reading intervention and detected improvements in emotional regulation among children with ADHD, providing evidence that measurable intervention-related changes can emerge over a relatively short period. The present 8-week protocol falls within the 4- to 12-week range used in these related AI-assisted interventions involving children with ADHD. We selected Doubao AI as the AI chatbot, which supported sound input and accurate transcribed conversation automatically (Miao et al., 2026; Yuan et al., 2026).

The RAs were also required to take a note check by using the checklist (see Appendix I) during each intervention. After each intervention, the RAs were required to submit the notes to the research team on time to ensure the effective supervision of the intervention. After the 8-week intervention, all the children underwent posttests using the same language measures and rhythm discrimination employed in the pretests to assess the effects of intervention.

### Research materials

The rhythm-related materials and phonological-awareness-related materials were used in the current study. Before the intervention, we invited 5 experts in music education and language learning to evaluate the appropriate of the given materials by using a 5-point Likert scale; any different opinions were fully discussed. Finally, all the materials used in the current study were scored over 4 points across the 5 experts.

#### Rhythm-related materials

The rhythm-related materials were developed to support the participating children’s perceptions of temporal regularity, beat, syllabic timing and rhythmic grouping in spoken Mandarin. These materials included auditory beat patterns, rhythmic chants, clapping and tapping prompts, syllable-to-beat mapping activities and rhythm-supported word repetition exercises. For example, when hearing the two-syllable word 苹果 (/ping 2 guo 3/, ‘apple’), the children were guided to produce two claps, with each clap corresponding to one syllable. This activity was intended to strengthen their awareness that spoken words can be segmented into smaller syllabic units. Similarly, rhythmic chants were used to present target words in a temporally regular format. For instance, the children might hear and repeat /mao 1/, /bao 1/, /shu 1/ in a rhythmic chant before identifying the word that did not rhyme with the others.

#### Phonological awareness-related materials

The PA-related materials targeted four components of Mandarin PA across syllable, rhyme, tone and phoneme-level awareness, matching the characteristics of Mandarin Chinese across syllables, rimes, lexical tones and phoneme-level units. The PA materials consisted of orally presented Mandarin words or syllables, picture prompts, practice items, test-like training items, corrective feedback scripts and child response sheets.

The AI chatbot presented the materials orally to the children with ADHD who then responded verbally. Words with highly unfamiliar meanings or those that were used with low frequency were avoided to reduce vocabulary-related difficulties, as confirmed by the aforementioned 5 experts. The syllable-awareness materials included Mandarin two- and three-syllable words familiar to young children. The activities required the children to listen to a word, repeat it, segment it into syllables, identify the first or last syllable or delete a target syllable. For example, when hearing “苹果/ping 2 guo 3/(apple)”, the children were asked to say what remained after deleting /píng 2/, with /guo 3/ as the correct response.

The rhyme-awareness materials included sets of three orally presented Mandarin syllables or words. In each set, two items shared the same rime, whereas one item had a different one. The children were asked to identify the item that did not rhyme with the other two. For example, the children heard /meng 1/, /beng 1/ and /lu 1/, then they were asked which one sounded different at the end. The correct response would be /lu 1/, because /meng 1/ and /beng 1/ shared the rime /eng/.

The tone-awareness materials consisted of Mandarin syllables with the same segmental structure but different lexical tones. The children listened to three syllables in each item and were asked to identify the syllable with a different tone. For example, they heard /ma 1/, /ma 1/ and /ma 3/, then they were asked which syllable had a different tone. The correct response was the third syllable /ma 3/.

The phoneme-level-awareness materials focused primarily on initial sound identification and initial phoneme deletion. Because phoneme-level awareness is relatively difficult for young Mandarin-speaking children, the training items progressed from easier initial-sound identification tasks to more demanding deletion tasks. For example, children heard /ma 1/and were asked to identify the first sound, with /m/ as the correct response. In a deletion item, children heard /ma 1/ and were asked what remained after removing /m/, with /a 1/ as the correct response.

Additionally, the materials used in the RPA and PAO group shared the same Mandarin target words, syllables, PA components and practice trials. The key difference was the mode of presentation. In the RPA materials, the children with ADHD completed the PA activities with rhythmic support, including beat patterns, clapping, tapping, rhythmic chanting and syllable-to-beat mapping. In the PAO materials, the same PA activities were presented through ordinary oral instruction without rhythm-based support. For example, both groups practiced syllable deletion using the word ‘苹果/ping 2 guo 3/ apple’. In the RPA materials, the children first clapped twice while saying /ping 2/ and /guo 3/ and then deleted /ping 2/. For materials used in PAO group, children listened to the same word and completed the same deletion task without clapping or rhythmic chanting. Similarly, both groups practiced rhyme awareness using item sets, such as /mao 1/, /bao 1/ and /shu 1/, but only the RPA materials presented the words in a rhythmic chant.

### Intervention condition

#### Rhythm-based PA intervention group

The children in the RPA group received an 8-week AI-supported rhythm-enhanced PA intervention. The intervention included two 30-min sessions per week, resulting in 16 sessions in total. Each session was delivered by the AI chatbot, while a trained RA provided technical and non-instructional behavioural support only. Specifically, the RA operated the device, used standardised non-instructional reminders to redirect the child’s attention to the chatbot when necessary and recorded implementation fidelity. The RA did not explain or rephrase instructional content, demonstrate task actions, provide hints or corrective feedback, evaluate the child’s responses or otherwise scaffold task completion. The intervention targeted four components of Mandarin PA across syllable awareness, rhyme awareness, tone awareness and phoneme-level awareness. Rhythm was embedded into each PA activity through beat perception, rhythmic chanting, clapping, tapping and syllable-to-beat mapping. For example, when learning syllable awareness, the children listened to a spoken word, repeated it rhythmically, clapped once for each syllable and then completed a syllable-deletion task. When learning rhyme awareness, the children listened to three words presented in a rhythmic chant and identified the word that did not share the same rime. When learning tone awareness, the children listened to syllables with different lexical tones and were guided to attend to pitch and rhythmic contours. When learning phoneme-level awareness, the children practiced identifying or deleting initial sounds after rhythmically repeating the target syllables. The AI chatbot delivered all instructional content and scaffolding, including oral instructions, target words, instructions for clapping, tapping and other task actions, rhythmic cues, task pacing and corrective feedback. The RA’s involvement remained limited to technical support, standardised attention redirection and fidelity recording.

#### PA intervention only group

The children in the PAO group received an 8-week AI- supported PA intervention with the same dosage as the RPA intervention group. The intervention was also delivered by the AI chatbot, with a trained RA providing the same technical and non-instructional behavioural support described for the RPA group. The RA did not provide any instructional explanations, demonstrations, hints, corrective feedback or evaluation of the children’s responses. The children in the PAO group received explicit training in the same four components of Mandarin PA across syllable awareness, rhyme awareness, tone awareness and phoneme-level awareness. The target words, number of trials, task order, feedback structure and session length were matched with those used in the RPA group. The only difference between the PAO and RPA groups was that the former completed the PA activities through ordinary oral instruction, without rhythmic chanting, beat-based clapping, tapping or syllable-to-beat mapping. For example, in syllable-awareness activities, the children listened to a target word and were asked to segment or delete a syllable without clapping to the syllabic beat. In the rhyme-awareness activities, they listened to three orally presented words and selected the word that did not rhyme with the others. In the tone-awareness activities, the children identified the syllable with a different lexical tone. Finally, in the phoneme-level awareness activities, they identified or deleted the initial sound from orally presented syllables.

#### Control group

The children with ADHD who were assigned to the control group received an 8-week AI- supported oral storybook listening activity program with the same frequency as the two intervention groups, two 30-min sessions per week for 8 weeks. The control activities were delivered by the AI chatbot, with a trained RA providing the same technical and non-instructional behavioural support used in the two intervention groups. The RA did not explain the story content, ask additional instructional questions, provide feedback on the children’s responses or otherwise scaffold their understanding of the stories. However, the control group did not receive any explicit PA related training, rhythm-based activities, beat synchronisation, clapping, syllable-to-beat mapping, rhyme judgment, tone discrimination or phoneme-deletion practice. After the posttest, the children in the control group were offered access to the RPA training materials as an ethical compensation.

### Data analysis

Chi-square and ANOVA tests were conducted to determine the presence of significant differences across demographic factors, language task and rhythm discrimination performances prior to the intervention. To evaluate intervention effects, we conducted separate repeated-measures general linear models (GLMs) for each outcome measure. Time (pretest vs. posttest) was entered as the within-subjects factor, and group (RPA vs. PAO vs. control) was entered as the between-subjects factor. Gender was dummy-coded (0 = boys, 1 = girls) and included as a categorical between-subjects factor. Age, nonverbal intelligence, working memory, and receptive vocabulary were entered as continuous covariates. The homogeneity-of-regression-slopes assumption was examined by testing the interactions between group and each continuous covariate, and no statistically significant group × covariate interactions were identified. The primary effect of interest was the time × group interaction. Furthermore, the effect sizes were measured using partial eta-squared ( $\eta_{p}^{2}$ ), with $\eta_{p}^{2}$ = 0.01, 0.06 and 0.14 were retained only as descriptive reference points for small, medium and large effects, respectively (Cohen, 1988). These conventional thresholds were used only as descriptive reference points. Because $\eta_{p}^{2}$ is design-dependent in repeated-measures models that include covariates, the effect-size estimates were interpreted alongside the unstandardized group means and adjusted mean differences rather than on the basis of these thresholds alone (Lakens, 2013). Additionally, Bonferroni correction was applied to post hoc pairwise comparisons to control for multiple testing.

### Fidelity check

To assess implementation fidelity, the RAs completed a structured fidelity checklist immediately after each intervention session. Specifically, the fidelity check determined whether: (a) the session lasted approximately 30 min; (b) the planned activities for the corresponding session were completed; (c) the AI educator delivered the scripted instructions, target words, pacing cues and corrective feedback; (d) the RA’s involvement remained limited to device operation, standardised attention redirection, procedural maintenance and fidelity recording; (e) the RA did not explain or rephrase instructional content, demonstrate task actions, provide hints or corrective feedback, evaluate children’s responses or otherwise scaffold task completion; (f) the children remained engaged and responded to the major learning tasks; and (g) no major technical problems occurred during the session. For the RPA group, additional fidelity indicators assessed whether rhythmic cues, clapping/tapping activities, rhythmic chanting and syllable-to-beat mapping were implemented as planned. Fidelity was coded dichotomously for each session item. For each domain, fidelity was calculated as the percentage of completed checklist items across all eligible sessions (Appendix II). The results showed that all groups performed the expected activities.

## Results

### Descriptive analysis

The performances of the children in the RPA, PAO and control groups at pre- and posttests are presented in Table 1. Baseline analysis showed that similar baselines were found across the three groups (*p*s > 0.10). Meanwhile, z scores showed that no outliers scores were included in the current database (within ±3 SD), thereby indicating the absence of outliers in the current study (Hodge and Austin, 2004).

**Table 1:** Descriptive information of children with ADHD between pretest and posttest.

Group	Variables	Pre_Mean	Pre_SD	Post_Mean	Post_SD
RPA (39 boys, 10 girls)	Age	5.55	0.3		
	Raven	24.27	3.9		
	RPR	50.23	14.05		
	WM	8.92	2.23		
	RV	36.63	4.17		
	CR	18.35	6.81	31.69	9.54
	PA_syllable	8.35	2.31	12.39	2.37
	PA_rhyme	4.74	1.5	7.31	1.58
	PA_tone	4.86	1.73	6.51	1.89
	PA_phoneme	5.78	2.09	8.37	2.58
	RD	10.57	2.08	13.82	2.4
PAO (34 boys, 14 girls)	Age	5.46	0.26		
	Raven	23.46	4.01		
	RPR	48.83	16.52		
	WM	8.35	2.13		
	RV	35.96	4.35		
	CR	18.96	9.01	24.77	9.73
	PA_syllable	7.5	2.04	10.67	2.35
	PA_rhyme	4.35	1.45	5.77	1.72
	PA_tone	5	1.52	5.96	1.76
	PA_phoneme	5.06	2.06	7.17	2.22
	RD	10.33	2.44	10.71	2.74
Control (39 boys, 10 girls)	Age	5.53	0.31		
	Raven	24.55	4.31		
	RPR	50.80	17.25		
	WM	8.65	2.32		
	RV	36.67	4.46		
	CR	18.27	7.77	25.49	10.99
	PA_syllable	8.16	2.68	8.9	2.99
	PA_rhyme	4.47	1.39	4.76	1.79
	PA_tone	5.02	1.56	5.04	2.01
	PA_phoneme	5.27	2.33	5.76	2.83
	RD	10.18	2.3	10.53	2.95

RPR = Raven percentile rank, WM = working memory, RV = receptive vocabulary, CR = character reading, RD = rhythm discrimination.

Table 1 also showed that the RPA group improved from 8.35 to 12.39 on the 16-item syllable-awareness measure, from 4.74 to 7.31 on the 10-item rhyme-awareness measure and from 10.57 to 13.82 on the 20-item rhythm-discrimination measure. The corresponding observed gains were 4.04, 2.57 and 3.25 points, whereas the control-group gains were 0.74, 0.29 and 0.35 points, respectively. For character reading, the observed gain was 13.34 characters in the RPA group and 7.22 characters in the control group.

*RQ1:* Do the two PA intervention conditions produce greater gains than the oral-storybook control condition within the shared delivery model?

Adjusted repeated-measures GLMs were conducted separately for character reading, syllable awareness, rhyme awareness, tone awareness, phoneme-level awareness, and rhythm discrimination. Each model included time as the within-subjects factor, group and gender as categorical between-subjects factors, and age, nonverbal intelligence, working memory, and receptive vocabulary as continuous covariates. Significant time effects were observed for all six outcomes (*p*s < 0.001, $\eta_{p}^{2}$ = 0.46–0.80). Significant time × group interactions were also found for all six outcomes (*p*s < 0.001, $\eta_{p}^{2}$ = 0.11–0.53; Table 2), indicating that pretest-to-posttest changes differed across the three groups.

**Table 2:** Time × Group interaction effects across RPA, PAO, and control groups.

Variables	Time effect		Time × Group		
	*F*	*η_p_* ^2^	*F*	*η_p_* ^2^	Comparison
CR	122.93^***^	0.46	8.51^***^	0.11	RPA > PAO = Control
PA_syllable	566.81^***^	0.80	79.59^***^	0.53	RPA > PAO > Control
PA_rhyme	366.29^***^	0.72	79.13^***^	0.53	RPA > PAO > Control
PA_tone	148.76^***^	0.51	43.55^***^	0.38	RPA = PAO > Control
PA_phoneme	341.69^***^	0.71	46.40^***^	0.39	RPA = PAO > Control
RD	141.80^***^	0.50	75.22^***^	0.51	RPA > PAO = Control

^***^ *p* < 0.001; η_p_^2^ = partial eta-squared; WM = working memory, RV = receptive vocabulary, CR = character reading, RD = rhythm discrimination.

The results of simple effect analysis showed that the RPA group had significantly higher scores than the control group across all selected measures (*p*s < 0.01). The PAO group had significantly higher scores than the control group in terms of PA-related skills (*p*s < 0.05). However, the simple effect analysis showed that the PAO group had similar scores with the control group in terms of character reading (mean difference = 0.72, *p* = 0.98) and rhythm discrimination (mean difference = 0.18, *p* = 0.98). These findings indicate that, within the shared AI-supported and RA facilitated interactive teaching model, the two PA intervention conditions produced greater gains in PA-related skills than the oral-storybook control condition.

*RQ2:* Does rhythm enhancement provide additional benefits beyond PA-only intervention within the shared delivery model?

The simple effect analysis showed that the RPA group had higher scores than the PAO group across character reading (mean difference = 6.92, *p* = 0.003), syllable awareness (mean difference = 1.72, *p* = 0.004), rhyme awareness (mean difference = 1.54, *p* < 0.001) and rhythm discrimination (mean difference = 3.11, *p* < 0.001). The RPA group had similar scores with the PAO group in tone awareness (mean difference = 0.55, *p* = 0.39) and phoneme-level awareness (mean difference = 1.20, *p* = 0.07). These findings suggest that, relative to PAO intervention, RPA intervention produced additional benefits in character reading, syllable awareness, rhyme awareness and rhythm discrimination, but not in tone awareness or phoneme-level awareness.

## Discussion

The present study examined whether an AI- supported RPA intervention could improve early reading and PA-related skills in children with ADHD and whether this RPA approach would produce additional benefits beyond a typical AI- supported PAO intervention. Two major findings were obtained. First, both the RPA and PAO groups showed greater gains than the control group in PA-related skills, indicating that PA intervention delivered through AI-supported and RA-facilitated interactive teaching model can support PA development in children with ADHD. Second, the RPA group outperformed the PAO group across character reading, syllable awareness, rhyme awareness and rhythm discrimination, although the two intervention groups showed similar developments in tone awareness and phoneme-level awareness.

The relatively large time × group interaction effect sizes observed after the 8-h intervention should be interpreted cautiously. In repeated-measures designs, $\eta_{p}^{2}$ is affected by features of the research design, including within-participant correlations and the inclusion of covariates. It is therefore most appropriately compared across studies using similar designs and should not be directly equated with standardised mean differences reported in meta-analyses using different analytical models (Olejnik and Algina, 2003; Lakens, 2013). More specifically, the largest value ( $\eta_{p}^{2}$ = 0.53) is an omnibus model-based estimate of the time × group interaction across the RPA, PAO and control conditions. It reflects the overall divergence in pretest-to-posttest changes across the three groups, rather than the unique additional effect of rhythm enhancement, and should not be interpreted as meaning that the intervention remediated 53% of the children’s PA difficulties.

A further consideration is the similarity between the intervention activities and the outcome measures. Transfer is generally considered more proximal when the training and assessment tasks share similar content, procedures and processing demands (Barnett and Ceci, 2002). In the present study, both RPA and PAO intervention groups explicitly and repeatedly practised syllable segmentation and deletion, rhyme discrimination, tone discrimination and initial-phoneme identification or deletion, whereas the control group received AI-supported oral-storybook activities without direct practice of these phonological operations. Because the immediate PA posttests assessed the corresponding phonological operations, the relatively large PA effects may therefore partly reflect near transfer from repeated, explicit and task-specific PA practice. This interpretation is consistent with evidence that direct PA instruction produces particularly strong effects on PA outcomes themselves (Ehri et al., 2001). It also distinguishes the present intervention from studies examining the transfer of general music training to literacy-related outcomes, for which the overall effect on PA has generally been smaller (Gordon et al., 2015). Similarly, Ahokas et al.’s (2025a, 2025b) intervention study differed from the present study in its participant population, training content, delivery format and outcome measures. Its training schedule should therefore not be treated as establishing a directly applicable minimum dosage for explicit PA intervention in kindergarten children with ADHD.

The raw scores reported in Table 1 provide a more concrete interpretation of the practical magnitude of the findings. The RPA group improved from 8.35 to 12.39 on the 16-item syllable-awareness measure, from 4.74 to 7.31 on the 10-item rhyme-awareness measure and from 10.57 to 13.82 on the 20-item rhythm-discrimination measure. The corresponding observed gains were 4.04, 2.57 and 3.25 points, whereas the control-group gains were 0.74, 0.29 and 0.35 points, respectively. For character reading, the observed gain was 13.34 characters in the RPA group and 7.22 characters in the control group. These values are descriptive raw-score changes rather than covariate-adjusted estimates, but they provide a more intuitive indication of the practical magnitude of the observed changes. Accordingly, the findings indicate clear immediate and task-specific improvements, but they should not be interpreted as showing that 8 h of intervention fully remediated PA or reading difficulties. Chan et al. (2023) found that reading interventions that included at least 30 h of training and targeted decoding or phonemic awareness met all benchmarks for classification as a Level 1 (Well-Established) Evidence-Based Practice with Strong Research Support for children with ADHD. This classification criterion does not mean that interventions shorter than 30 h cannot produce statistically significant or immediate task-specific gains. Rather, it indicates that the present 8-h study should not be interpreted as establishing comprehensive, durable or well-established remediation comparable with the evidence available for substantially longer reading interventions. Moreover, because the present study did not include a typically developing comparison group or age-standardised outcome scores, it cannot determine whether the children with ADHD reached the PA or reading level expected for typically developing peers.

### AI-supported PA intervention in children with ADHD

The results partially kept consistent with previous empirical designs, which showed that AI chatbot-supported language interventions significantly enhanced the language development of children with ADHD (Dong et al., 2026; Miao et al., 2026), and with the broader literature on AI-supported intervention delivery in K–12 education, learning-disability support and pediatric contexts (Klapow et al., 2024; Létourneau et al., 2025; Paglialunga and Melogno, 2025; Xu et al., 2025). However, because all three conditions in the present study used the same AI-supported and RA-facilitated interactive teaching model, the findings do not demonstrate that AI was more effective than human-led instruction. Instead, the results show that explicit and systematic PA activities could be implemented within this shared delivery model and that adding rhythmic support produced specific benefits beyond PA-only instruction. These findings are also consistent with previous PA research showing that explicit and systematic instruction can improve children’s phonological and reading-related outcomes (Chan et al., 2023; Ehri et al., 2001; Ruan et al., 2024; Shen et al., 2025; Zhang et al., 2026).

Regarding syllable awareness, this result may be explained by the fact that syllable-level tasks were highly compatible with AI- supported instruction. In the intervention conducted in the present study, the children repeatedly heard familiar Mandarin two- or three-syllable words, repeated them, segmented them, and then identified or deleted target syllables. The AI chatbot supported the standardised delivery of this process by presenting the same target words, providing consistent task prompts across children and delivering corrective feedback immediately after children’s responses. These features may have helped the children with ADHD maintain attention to the syllabic structure of spoken words and reduced variation in instructional prompting. Because Mandarin syllables are perceptually salient units, repeated and standardised exposure to word segmentation and syllable deletion may have made the internal syllabic structure of words more explicit (Dong et al., 2020; Shu et al., 2008). Furthermore, the fidelity checklist coding confirmed that syllable-awareness activities were implemented in 98% of eligible intervention sessions and that the AI chatbot delivered target words, standardised prompts and corrective feedback with 97% accuracy across all trials. Therefore, the improvement in syllable awareness may reflect the benefit of repeated, structured and standardised practice with salient Mandarin phonological units.

Regarding rhyme awareness, the results may be explained by the consistent presentation of word sets for phonological comparison within the AI-supported intervention. The rhyme-awareness tasks required the children in this study to compare the final sound patterns of several orally presented words and identify the item that did not share the same rime. Variations in pronunciation, pausing, emphasis or feedback may influence how children perceive similarities among words (Luthra, 2024; Quam and Creel, 2021). In the present AI-supported intervention, the chatbot presented the rhyme items in a standardised format while providing consistent prompts and feedback across sessions. This consistency may have reduced irrelevant variability in the auditory input and allowed the children to focus more directly on the rime structure of the words. For children with ADHD, who may be easily distracted by changes in voice, pace or presentation style, this stable delivery format may have helped them compare word endings more reliably (Kofler et al., 2013). Additionally, the fidelity coding further indicated that rhyme-awareness activities were completed in 96% of targeted sessions and that AI pronunciation and target-word presentation for rhyme contrasts remained 99% consistent without unintended variation. Therefore, the gains in rhyme awareness may reflect the controlled auditory comparisons and repeated practice with rime contrasts provided within the intervention.

Regarding tone awareness, the observed improvements may be explained by the consistent presentation of Mandarin tone contrasts within the intervention. The tone-awareness tasks required the children to detect differences in lexical tone while holding the segmental structure constant. This is cognitively demanding because they must attend to pitch contour rather than segmental differences or word meaning. In the AI-supported PA intervention in the current study, the children repeatedly heard tone-contrast items and were prompted to identify which syllable had a different tone. The AI-supported delivery format may have supported this process through consistent wording, a stable task structure and repeated feedback. For children with ADHD, such predictability may reduce task confusion and help them focus on the relevant acoustic dimension. Furthermore, the intervention checklist revealed that tone-awareness activities were delivered as designed in 95% of the sessions and that the children followed AI prompts for tone discrimination tasks with 92% engagement throughout the intervention. Therefore, the improvement in tone awareness may have resulted from repeated and explicit attention to tonal differences in Mandarin.

Regarding phoneme-level awareness, the observed gains may be related to the explicit and predictable instruction provided for this relatively difficult PA component. Phoneme-level awareness is less perceptually salient than syllable or rhyme awareness for young Mandarin-speaking children, because Mandarin speech is naturally organised around syllables. Therefore, the children may need more explicit prompts to identify or delete initial sounds. In the present intervention, the participating children were repeatedly guided to attend to initial sounds, identify target phonemes and complete phoneme-deletion tasks. Because the task format, instructions and feedback were kept stable across sessions, they were able to gradually familiarise themselves with the phonological operation itself rather than repeatedly adapting to new instructional wording. This may be particularly helpful for children with ADHD, as predictable routines can reduce cognitive load and help them maintain attention during difficult sound-manipulation tasks. Additionally, the fidelity data verified that phoneme-level awareness activities were implemented in 94% of the sessions and that the RAs provided no additional instructional explanations in 99% of trials, thereby supporting adherence to the standardised intervention protocol. Thus, the improvement in phoneme-level awareness may reflect the repeated, explicit and standardised guidance provided during phoneme identification and manipulation.

### The added value of RPA intervention over PAO intervention

The results of the present study are consistent with prior rhythm-reading research suggesting that rhythm and prosodic sensitivity are related to children’s PA and early reading (Gutiérrez-Palma et al., 2026; Harrison et al., 2018; Holliman et al., 2008). The stronger effect of RPA on syllable awareness might be explained by the direct match between rhythm-based activities and syllable segmentation (Corriveau and Goswami, 2009; Goswami, 2011; Thomson et al., 2013). The children in the RPA group did not simply hear a word and complete a syllable-deletion task; they were guided to connect each syllable with a beat through clapping, tapping, rhythmic repetition and syllable-to-beat mapping. For example, when they heard a two-syllable word such as *pingguo*, they clapped twice, with each clap corresponding to one syllable, before completing the syllable manipulation task. This may have made the internal syllabic structure of spoken words more concrete and perceptually salient (Goswami, 2011; Power et al., 2013).

For children with ADHD, such external temporal structure may be particularly useful because it reduces the demand to hold the whole spoken word in working memory while simultaneously segmenting it (Goswami, 2011; Thomson et al., 2013). Additionally, the fidelity checklist coding confirmed that syllable-to-beat mapping and beat-aligned clapping activities were implemented in 98% of the RPA sessions, whereas the PAO sessions showed 100% compliance with the protocol by providing no rhythm-based syllabic activities. Thus, RPA may have improved syllable awareness more than PAO because rhythm converted syllable segmentation from a purely auditory-verbal operation into an auditory-motor and temporally organised activity (Corriveau and Goswami, 2009; Thomson et al., 2013).

The RPA advantage in rhyme awareness could be attributed to rhythmic chanting and temporal grouping (Harrison et al., 2018; Thomson et al., 2013). Rhyme-awareness tasks required the children to compare the final sound patterns of orally presented words and identify which item does not share the same rime. In the PAO group, the children completed this comparison through ordinary oral instruction. In the RPA group, however, the same word sets were presented through rhythmic chants before the children made rhyme judgments. Here, the rhythmic chanting may have stabilised the timing of the word sequence and helped the children attend to repeated sound endings (Goswami, 2011; Power et al., 2013). This explanation is consistent with previous findings showing links between speech rhythm, prosodic sensitivity, PA and word reading (Harrison et al., 2018; Holliman et al., 2008; Power et al., 2013). Additionally, the intervention checklist verified that rhythmic chanting for rhyme contrasts was delivered in 97% of the RPA sessions, while rhyme-awareness activities in the PAO were completed without any rhythmic support in 99% of the trials. Therefore, the stronger rhyme-awareness gain in RPA may have occurred because the rhythm helped the children detect sound similarities across words, especially word-final phonological similarity (Goswami, 2011; Thomson et al., 2013; Wang and Chen, 2025).

The RPA advantage in rhythm discrimination might have also been due to the RPA intervention directly training rhythm-related processes through beat perception, clapping, tapping, rhythmic chanting and syllable-to-beat mapping, whereas the PAO did not include rhythm-based activities (Corriveau and Goswami, 2009; Thomson et al., 2013). Consequently, the RPA group repeatedly practiced attending to temporal regularity and rhythmic contrast, which likely transferred to the rhythm discrimination task (Gordon et al., 2015; Thomson et al., 2013). Furthermore, the rhythm discrimination task used nonspeech metronome-like tones and did not require the children to produce clapping or tapping responses, suggesting that the RPA effect was not merely a task-practice effect but may reflect improved sensitivity to rhythmic patterns. Additionally, the fidelity data confirmed that beat perception, rhythmic chanting and clapping/tapping exercises were implemented in 99% of the RPA sessions and that no rhythm-related activities were provided in any PAO sessions. The finding is also consistent with recent Chinese evidence showing that musical rhythm discrimination is associated with PA and reading-related abilities (Wang and Chen, 2025).

The stronger effect of RPA on character reading suggests that typical PAO training may mainly produce near-transfer effects on trained phonological tasks, while RPA training may be more likely to support far-transfer to early reading (Barnett and Ceci, 2002). In Chinese character reading, children need to connect visual character forms with spoken syllables and lexical meanings (Li et al., 2020; Shu et al., 2008). The checklist results showed that RPA integrated rhythmic cues with phonological practice in 96% of all intervention sessions and that the children maintained high engagement (93%) with rhythm-enhanced PA tasks throughout the 8-week program. In this sense, rhythm may have served as a bridge between PA practice and reading transfer (Ahokas et al., 2025a, 2025b; Gordon et al., 2015; Harrison et al., 2018). In particular, it did not merely make the activities more engaging, but reorganised phonological input in a way that made spoken units more stable and usable for early character reading (Goswami, 2011; Power et al., 2013).

One interesting finding is the similar development in tone awareness and phoneme-level awareness between the RPA and PAO groups. Regarding tone awareness, in the current study, the RPA and PAO groups received explicit tone-awareness practice through AI-supported instruction, target syllables and standardised feedback. Therefore, tone-awareness gains may have depended mainly on repeated exposure to tone contrasts and explicit attention to pitch contour, rather than on beat-based rhythmic support (Shu et al., 2008; Zhang et al., 2026). Additionally, the fidelity coding confirmed that tone-awareness activities were implemented with equal fidelity in the RPA (95%) and PAO (96%) sessions, and no rhythm-specific support was added to tone tasks in either condition. Rhythm can organise timing, grouping and syllabic structure, but lexical tone discrimination depends more directly on pitch contour and tonal category perception (Goswami, 2011; Shu et al., 2008).

Regarding phoneme-level awareness, this result may be explained by the developmental and linguistic difficulty of phoneme-level awareness in Mandarin-speaking children (Shu et al., 2008). Mandarin phonological structure makes syllables and rimes more perceptually salient than individual phonemes; therefore, phoneme-level manipulation may require more explicit and intensive training than syllable- or rime-level tasks (Ehri et al., 2001; Shen et al., 2025; Shu et al., 2008). In the present intervention, both the RPA and PAO groups practiced the same initial sound identification and deletion tasks using the same target items and feedback structure. Rhythm may have supported larger temporal units, such as syllables and rimes more directly than smaller phoneme-level units (Goswami, 2011; Power et al., 2013; Thomson et al., 2013). The checklist also verified that phoneme-level awareness activities were completed with identical fidelity (94% for RPA and 95% for PAO) and that rhythm was not integrated into phoneme-level tasks in either group. Therefore, the 8-week RPA intervention may have been sufficient to produce clear advantages in syllable and rhyme awareness. However, these were not strong enough to create a statistically significant advantage in phoneme-level awareness.

### Limitations and implications

This study has some limitations which should be addressed in future studies. First, this study only assessed immediate post-intervention effects. Therefore, it remains unclear whether the observed gains in PA, rhythm discrimination and character reading represent stable developmental improvement or short-term practice effects. The close correspondence between the PA training activities and the immediate PA posttests may have contributed to the magnitude of the proximal effects. Second, the fidelity check mainly focused on whether the intervention was implemented as planned, rather than on the quality of the children’s learning processes. Although the checklist confirmed session duration, activity completion, AI prompt delivery, facilitator adherence, child engagement and technical stability, it did not capture detailed process-level indicators, such as the children’s response accuracy during training, attention fluctuation, number of repeated prompts or facilitator support. Because children with ADHD may vary greatly in terms of attention and task persistence, future studies should collect fine-grained process data to explain why some children benefit more than others. Third, the intervention was AI-supported and included non-instructional RA facilitation. The AI chatbot delivered all instructional content and scaffolding, including instructions, target words, instructions for task actions, pacing cues, rhythmic cues and corrective feedback. The trained RAs were limited to device operation, standardised attention redirection and fidelity recording and did not provide any instructional scaffolding. Nevertheless, the RAs’ presence and behavioural support may still have influenced the children’s engagement and task persistence. Accordingly, the present design cannot isolate the independent contribution of AI, determine whether the same outcomes would be obtained without RA facilitation or establish whether AI-supported intervention is more effective than human-led intervention. Future studies should compare AI-only intervention, AI-supported intervention with non-instructional RA facilitation and human-led intervention to clarify the contribution of each delivery component. Fourth, the current study did not include an age-matched typically developing comparison group, and the outcome measures were analysed as raw scores rather than age-standardised, norm-referenced scores. Therefore, the present study cannot determine whether the post-intervention performance of children with ADHD reached the level expected for typically developing children. Future studies should include an age-matched typically developing benchmark group or validated age-standardised scores to determine whether the intervention narrows the developmental gap. Next, although the participants were randomly allocated after stratification by gender, nonverbal intelligence and working memory were not used as matching or stratification variables in the original allocation procedure. Given the potential relevance of these cognitive abilities to PA learning and intervention response, incorporating them into a restricted randomisation procedure might have further reduced the possibility of chance baseline imbalance. The baseline analyses did not detect statistically significant differences in these measures across the three groups, and both nonverbal intelligence and working memory were included as covariates in the adjusted analyses. Future studies could assess these variables before allocation and incorporate them into the stratified randomisation procedure. Finally, the exclusive inclusion of children with ADHD and no identified comorbidities may limit the generalizability of our findings. In clinical and community settings, a substantial proportion of children with ADHD present with co-occurring neurodevelopmental, psychiatric or behavioural conditions, which may moderate intervention responses.

This study also has several implications. First, the results suggest that AI-supported delivery can be feasibly incorporated into structured PA intervention when trained human facilitation is available, especially when the target group (i.e., children in this case) require repeated practice, consistent prompts and immediate feedback. Second, RPA activities may be more useful than typical PAO training when the intervention goal is to improve not only PA skills but also character reading and rhythm discrimination. This is based on the fact that the RPA group in this study showed greater gains than the PAO group in character reading, syllable awareness, rhyme awareness and rhythm discrimination. From a practical perspective, kindergarten-based PA intervention for children with ADHD may benefit from embedding rhythmic chanting, clapping, tapping and syllable-to-beat mapping into AI-supported PA activities. In addition, the study results showed that RPA training may provide temporal scaffolding for children’s processing of syllabic and rhyming structures. At the same time, the findings suggest that rhythm effects are domain-specific, as RPA produced additional benefits for syllable awareness and rhyme awareness, but not for tone awareness or phoneme-level awareness. More broadly, this study extends AI-supported language intervention research by showing the feasibility of using AI as a standardised delivery component within a RA-facilitated interactive teaching intervention model.

## Conclusion

This study examined whether an AI-supported rhythm-enhanced PA intervention could improve early reading, PA-related skills and rhythm discrimination in Mandarin-speaking children with ADHD and whether rhythm-enhanced PA intervention would produce additional benefits beyond typical AI- supported PA intervention. The results suggest that rhythm provided an important temporal and attentional scaffold for PA learning in children with ADHD. Moreover, the study advances the literature by integrating rhythm-based language intervention, ADHD-focused intervention and AI-supported instruction into a unified intervention framework. The present study suggests that AI can serve as a standardised delivery component within a structured and human-facilitated intervention model. However, because AI-only and human-led comparison conditions were not included, the independent contribution of AI cannot be determined from the present findings.

## Data Availability

The raw data supporting the conclusions of this article will be made available by the authors, without undue reservation.
