# Supplementary material for: Does artificial intelligence-supported rhythm-enhanced phonological awareness training improve early reading in children with attention-deficit/hyperactivity disorder? A randomised controlled intervention study
Source: Front Psychol. 2026 Sep 3;17:1906514. doi: 10.3389/fpsyg.2026.1906514 (PMC13582397; doi:10.3389/fpsyg.2026.1906514)
Supplement: Supplementary file 1 [file supplementary_file_1.docx]

*Appendix I*

*Checklist for Intervention*

| *因素(Domain)* | *核查项目 (Checklist Item)* |
| --- | --- |
| *干预剂量 (Dosage)* | *每次干预持续28–32分钟 (Session lasted 28–32 minutes)* |
| *干预剂量 (Dosage)* | *儿童参加了完整的干预活动 (Child attended the full session)* |
| *干预剂量 (Dosage)* | *完成了计划中的活动数量 (Planned number of activities was completed)* |
| *AI执行 (AI Delivery)* | *AI提供了脚本化的口头指令 (AI delivered scripted oral instructions)* |
| *AI执行 (AI Delivery)* | *AI正确呈现了目标词/音节 (AI presented the target words/syllables correctly)* |
| *AI执行 (AI Delivery)* | *AI提供了标准化的纠正反馈 (AI provided standardized corrective feedback)* |
| *音韵意识内容 (PA Content)* | *实施了音节意识活动 (Syllable-awareness activity was implemented)* |
| *音韵意识内容 (PA Content)* | *实施了押韵意识活动 (Rhyme-awareness activity was implemented)* |
| *音韵意识内容 (PA Content)* | *实施了声调意识活动 (Tone-awareness activity was implemented)* |
| *音韵意识内容 (PA Content)* | *实施了音位层面意识活动 (Phoneme-level awareness activity was implemented)* |
| *节奏内容 (Rhythm Content)* | *提供了节拍/节奏提示 (Beat/rhythm cues were provided)* |
| *节奏内容 (Rhythm Content)* | *实施了拍手/敲击活动 (Clapping/tapping activity was implemented)* |
| *节奏内容 (Rhythm Content)* | *实施了节奏吟诵活动 (Rhythmic chanting was implemented)* |
| *节奏内容 (Rhythm Content)* | *实施了音节—节拍对应活动 (Syllable-to-beat mapping was implemented)* |
| *条件区分 (Differentiation)* | *未提供节奏型活动 (No rhythm-based activity was provided)* |
| *条件区分 (Differentiation)* | *未提供显性音韵意识教学 (No explicit PA instruction was provided)* |
| *研究助理角色 (RA Role)* | *研究助理未解释或改述教学内容，未示范任务动作，未提供提示或纠正反馈，也未评价儿童的回答。 (RA did not explain or rephrase instructional content, demonstrate task actions, provide hints or corrective feedback, or evaluate the child’s responses.)* |
| *研究助理角色 (RA Role)* | *研究助理的支持仅限于设备操作、标准化注意力重定向、流程维护和忠实度记录。 (RA support was limited to device operation, standardised attention redirection, procedural maintenance and fidelity recording.)* |
| *儿童参与 (Child Engagement)* | *儿童在大多数时间跟随AI提示 (Child followed the AI prompts most of the time)* |
| *儿童参与 (Child Engagement)* | *儿童对主要任务作出口头或行为反应 (Child gave verbal or behavioral responses to major tasks)* |
| *技术执行 (Technical Fidelity)* | *AI音频/输出功能正常 (AI audio/output functioned normally)* |
| *技术执行 (Technical Fidelity)* | *未发生重大设备中断 (No major equipment interruption occurred)* |
| *污染控制 (Contamination)* | *儿童未接触到其他条件的活动内容 (Child was not exposed to activities from another condition)* |
| *记录说明 (Documentation)* | *记录了任何偏离干预方案的情况 (Any deviation from the protocol was recorded)* |

Appendix II

*Overview of Coding Results for Checklist*

| Fidelity indicator | RPA | PAO | Control |
| --- | --- | --- | --- |
| Session duration within 28–32 minutes | 0.96 | 0.95 | 0.97 |
| AI delivered scripted instructions, target items, and feedback as planned | 0.98 | 0.97 | 0.96 |
| Planned PA activities were completed | 0.97 | 0.96 | N/A |
| Syllable-, rhyme-, tone-, and phoneme-level PA activities were implemented as planned | 0.96 | 0.99 | N/A |
| Rhythm-enhanced activities were implemented as planned | 0.98 | N/A | N/A |
| No rhythm-based activities were provided | N/A | 0.99 | 1.00 |
| No explicit PA instruction was provided | N/A | N/A | 1.00 |
| Research assistants provided no additional instructional explanation | 0.99 | 0.98 | 0.99 |
| Children followed AI prompts and responded to major tasks | 0.94 | 0.93 | 0.92 |
| No major technical interruption occurred | 1.00 | 1.00 | 1.00 |

*Note*. RPA = rhythm-based phonological awareness intervention; PAO = phonological awareness-only intervention. N/A indicates that the indicator was not applicable to that condition. Percentages represent the proportion of eligible sessions in which each fidelity indicator was coded as implemented.
